# Factors Associated with Immunization Opinion Leadership among Men Who Have Sex with Men in Los Angeles, California

**DOI:** 10.3390/ijerph15050939

**Published:** 2018-05-08

**Authors:** Ian W. Holloway, Robert Bednarczyk, Vincent L. Fenimore, Cameron Goldbeck, Elizabeth Wu, Rebecca Himmelstein, Diane Tan, Laura Randall, Chelsea S. Lutz, Paula M. Frew

**Affiliations:** 1 Department of Social Welfare, UCLA Luskin School of Public Affairs, 3255 Charles E. Young Drive East, Los Angeles, CA 90095, USA; cgoldbeck@ucla.edu (C.G.); ElizabethWu@luskin.ucla.edu (E.W.); rlhimmel@luskin.ucla.edu (R.H.); diane.tan@ucla.edu (D.T.); 2 UCLA Center for AIDS Research, Los Angeles, CA 90095, USA; 3 Southern California HIV/AIDS Policy Research Center, Los Angeles, CA 90095, USA; 4 Hubert Department of Global Health, Rollins School of Public Health, Emory University, 1518 Clifton Road, Atlanta, GA 30322, USA; robert.a.bednarczyk@emory.edu (R.B.); vfenimo@emory.edu (V.L.F.); pfrew@emory.edu (P.M.F.); 5 Division of Infectious Diseases, Department of Medicine, Emory University School of Medicine, 1760 Haygood Road, Atlanta, GA 30322, USA; laura.randall@emory.edu (L.R.); chelsea.lutz@emory.edu (C.S.L.); 6 Emory Center for AIDS Research, 1518 Clifton Road, Atlanta, GA 30322, USA

**Keywords:** opinion leadership, MSM, peer health navigation, vaccine measurement, immunization acceptance, immunization assessment, vaccine promotion

## Abstract

We sought to identify the characteristics of men who have sex with men (MSM) who are opinion leaders on immunization issues and to identify potential opportunities to leverage their influence for vaccine promotion within MSM communities. Using venue-based sampling, we recruited and enrolled MSM living in Los Angeles (*N* = 520) from December 2016 to February 2017 and evaluated characteristic differences in sociodemographic characteristics, health behaviors, and technology use among those classified as opinion leaders versus those who were not. We also asked respondents about their past receipt of meningococcal serogroups A, C, W, and Y (MenACWY) and meningococcal B (MenB) vaccines, as well as their opinions on the importance of 13 additional vaccines. Multivariable results revealed that non-Hispanic black (aOR = 2.64; 95% CI: 1.17–5.95) and other race/ethnicity (aOR = 2.98; 95% CI: 1.41–6.29) respondents, as well as those with a history of an STI other than HIV (aOR = 1.95; 95% CI: 1.10–3.48), were more likely to be opinion leaders. MenACWY (aOR = 1.92; 95% CI: 1.13–3.25) and MenB (aOR = 3.09; 95% CI: 1.77–5.41) vaccine uptake, and perceived importance for these and seven additional vaccines, were also associated with being an opinion leader. The results suggest that the co-promotion of vaccination and other health promotion initiatives via opinion leaders could be a useful strategy for increasing vaccination among MSM.

## 1. Introduction

Although many studies have examined factors associated with immunization hesitancy, refusal, and acceptance decision-making, as well as immunization coverage that may cluster within networks, the factors associated with opinion leadership that drive vaccine attitudes and vaccine choices is an understudied phenomenon [1,2,3]. Opinion leadership is characterized as holding considerable social influence within a network or community, resulting in diffusion of thought, persuasion, and behavior of others [4,5]. In communities of men who have sex with men (MSM), there have been recent vaccine preventable disease (VPDs) outbreaks (i.e., meningitis) that have resulted in federal, state, and local health department immunization recommendations; however, immunization uptake observed among those surveyed is persistently low [6,7,8].

MSM communities are characterized by strong, dense social networks, often formed and maintained through technology [9], which can be valuable targets for immunization interventions [10]. A previous study assessed the five clusters of parental vaccine opinions identified by Gust and colleagues (2005) and suggested more comprehensive research to assess additional factors associated with the formation of positive opinion leadership was needed [11]. Thus, improving vaccine uptake through peer health influencers is an underutilized public health strategy that may offset many health and social threats including life-years lost, hospitalized days, outpatient visits, loss of productivity, and significant health costs [12,13,14]. 

Opinion leadership has—until now—largely been operationalized within the context of marketing and consumerism, or for specific health interventions such as HIV prevention among MSM. For example, marketing based on social networks use opinion leaders as diffusers within their networks to accelerate the uptake of a new service or product [15,16,17]. Similarly, peer-led interventions have been found to significantly increase HIV testing [18,19], knowledge seeking [19], and condom use [19,20,21,22] among MSM in high and middle income countries. These interventions have also been moved onto social media platforms, such as Facebook. For example, in a study by Young and colleagues (2014) MSM randomly assigned to a peer opinion leadership group on Facebook that focused on HIV prevention had lower self-reported risk and higher uptake of HIV home testing kits at the conclusion of a 12-week study [23].

Our project sought to examine the characteristics associated with opinion leadership for MSM vaccination in the context of an ongoing meningococcal outbreak in Southern California [24]. We assessed specific vaccine importance to understand what vaccines were important to opinion leaders and what demographic and behavioral characteristics were unique to opinion leaders. In addition, we analyzed technology and social media use to understand whether these venues may be appropriate channels for opinion leader immunization interventions. This work may be particularly useful in developing and prioritizing future vaccine messages that may be rapidly diffused through MSM communities.

## 2. Materials and Methods 

### 2.1. Data Collection

Data were collected from November 2016 through February 2017 as part of a larger project to understand MSM vaccination response in the context of an ongoing meningitis outbreak in Southern California [8,24]. Using venue-based convenience sampling, we were able to approach 2250 men about potential participation in the study. Recruiters asked a brief series of standardized questions to those who expressed interest and recorded responses on a tablet computer to assess their eligibility. Persons were eligible to enroll if they met the following eligibility criteria: (1) were at least 18 years old; (2) were assigned male sex at birth; (3) identified as male; (4) reported having sex with men in the past three months; (5) resided in Los Angeles County; (6) spoke English or Spanish; and (7) were able to provide informed consent. Eligible MSM were invited in person to take the 15–30-min survey (mean = 17.66 min) delivered by trained interviewers on tablet computers in field settings. All participants received a $50 cash incentive for completing the survey. The North Campus Institutional Review Board (IRB) at the University of California, Los Angeles reviewed and approved the study prior to survey implementation (IRB#16-001745); Emory University subsequently reviewed it as a secondary data analyses project (IRB#96595).

### 2.2. Measures

The outcome of interest was opinion leadership, which we defined using five survey items regarding the influence of one’s opinion on the vaccination of one’s peers. These survey items were: (1) “My opinion on vaccines does not seem to count when I talk with others”; (2) “I often influence other’s opinions about vaccines”; (3) “When they choose their vaccines, others do not turn to me for advice”; (4) “I often persuade others to get vaccinated”; and (5) “Others rarely come to me for advice about choosing vaccines”. Each item was scored on a five-point Likert scale of 1 to 5, representing “Strongly Disagree”, “Disagree”, “Neutral”, “Agree”, and “Strongly Agree” respectively. Together the standardized Cronbach’s alpha score was 0.67, suggesting moderate internal consistency between the survey items. Items 1, 3, and 5 were worded in the negative so scores were reverse-coded so that all item responses were in the positive. Opinion leadership was then created first by summing scores across each of these variables and then dichotomizing the score so that those who scored 20 or greater were considered to be opinion leaders and those who scored less than 20 were considered to be non-opinion leaders. This cut-off was chosen because it represented an overall opinion leadership score in the top quintile of the scale’s range. Additionally, 13.5% (*N* = 70) of respondents scored above 20 on the scale, which is consistent with literature describing opinion leaders as “innovators” or “early adopters” in the top 15% of Rogers’ Diffusion of Innovations curve [25]. 

We were interested in the predictive ability of 15 vaccination uptake and importance variables on opinion leadership. These variables were two vaccine uptake questions (yes/no/unsure) of meningococcal serogroups A, C, W, and Y (MenACWY) and meningococcal B (MenB), and 13 vaccine importance questions (yes/no/unsure) regarding MenACWY; MenB; human papillomavirus (HPV); hepatitis A (HAV); hepatitis B (HBV); influenza (flu); *Haemophilus influenzae* type b (Hib); measles, mumps, rubella (MMR); tetanus, diphtheria, pertussis (Td/Tdap); varicella; zoster; 13-valent pneumococcal conjugate (PCV13); and 23-valent pneumococcal polysaccharide (PPSV23). For the analysis, “no” and “unsure” were collapsed together. 

To evaluate vaccine confidence, we developed a 10-item MSM Vaccine Confidence Index (MSM VCI) score using items concerning confidence about vaccines and immunization receipt, following similar methodology used in the development of three parallel indices for parental vaccine confidence [26]. These survey items were identified from the 30-item vaccine confidence-related framework developed by the National Vaccine Advisory Committee (NVAC) and were classified as they corresponded to the trust-related constructs of the “Information Environment”, in their “Healthcare Provider”, and “Attitudes and Beliefs” [27]. Each item was scored on a five-point Likert scale from 1 to 5, representing “Strongly Disagree”, “Disagree”, “Neutral”, “Agree”, and “Strongly Agree”, respectively. The MSM VCI summary score was created by summing scores across each of these variables, resulting in a potential range of 10 to 50.

Participants were also asked about their age, race/ethnicity, education, income, employment status, health insurance, residential zip code, substance use, sexual risk behaviors (e.g., condomless receptive anal sex in the past six months), STI history, health promoting behaviors (e.g., multivitamin use and PrEP use among those who self-reported being HIV-negative), and HIV status. We used condomless receptive anal sex, age, and drug use to develop a risk score using previously established criteria [28]. 

Finally, we asked participants about their technology use. Three questions assessed frequency of individuals’ email, phone, and social media use. We also asked participants to specify the types of social networking websites (e.g., Facebook, Twitter) they had used in the past 30 days. Additionally, we asked if participants had ever used the internet to find vaccination information (yes/no) or to find where to get vaccinated (yes/no).

### 2.3. Statistical Analysis

We evaluated differences in sociodemographic characteristics, health indicators, and technology use among those classified as opinion leaders versus those who were not using bivariate and multivariable analyses. Variables were identified for the multivariable analysis based on significant bivariate results. To assess the predictive ability of the 15 vaccination uptake/importance items, we conducted separate logistic regressions to estimate the increase in the odds of opinion leadership for those who positively (“yes”) responded to the 15 items compared to those who did not (“no” or “unsure”). All statistical analyses were performed using SAS 9.3 (SAS Institute, Cary, NC, USA) [29], with significance defined as *α* = 0.05 

## 3. Results

### 3.1. Sociodemograghic Characteristics

Of the 2250 men approached during venue-based sampling, 749 were screened, of which 520 provided valid responses, for an overall response rate of 69%. Respondent sociodemographic characteristics are described in Table 1. Overall, 70 (13%) were classified as opinion leaders. Participants were young (M = 33.3, SD = 10.1) and racially/ethnically diverse: White (35.4%), Black/African American (15.1%), Hispanic (32.5%), and other race/ethnicity (17.0%). Half (51.3%) were college educated. One-fifth (21.1%) made less than $20,000 per year; the majority (88.3%) had some form of health insurance. Approximately one-third (34.0%) resided in West Hollywood, a gay enclave in Los Angeles. Most (89.9%) reported sex with men only, with over a quarter (27.8%) reporting six or more sexual partners in the past six months. Half (51.9%) reported condomless anal sex (CAS) within the same time frame. Nearly 12% (11.9%) were HIV-positive.

### 3.2. Bivariate and Multivariable Results

Opinion leaders and non-opinion leaders had similar sociodemographic characteristics, except for race/ethnicity. There were more Black/African American (21.7%) or other race/ethnicity (26.1%) opinion leaders compared to non-opinion leaders (14.1% and 15.4%) respectively (Table 1). There were greater percentages of participants living with HIV among opinion leaders (20.3%) compared to non-opinion leaders (10.6%). History of a sexually transmitted infection (STI) other than HIV was also associated with opinion leadership with 64.3% of opinion leaders having ever been diagnosed with an STI compared to 45.2% of non-opinion leaders. Additionally, among those who were HIV-negative, there was also twice as much reported PrEP use for opinion leaders (29.1%) compared to non-opinion leaders (15.9%). However, these characteristics did not significantly affect those classified as “high risk”; 71.2% of opinion leaders and 65.2% of non-opinion leaders were classified as “high risk”, though this difference was not significant (*p* = 0.34).

Technology use among sample participants was high and there were no significant differences in technology use between the two groups (Table 1). Both opinion leaders and non-opinion leaders, respectively, had high proportions of Facebook use (85.7% and 86.6%), daily email use (92.8% and 90.9%), daily phone use (98.6% and 98.9%), and daily social media use (95.3% and 89.4%). Additionally, the majority of opinion leaders and of non-opinion leaders reported using the internet to access information about vaccinations (64.3% and 57.6%); 51.4% of opinion leaders and 46.2% of non-opinion leaders reported using the internet to find the locations of vaccination-providers. 

We noted statistically significant differences in vaccination uptake, confidence, and importance. Vaccination uptake and importance tended to be higher for opinion leaders. Higher rates of uptake for both meningococcal vaccines were seen in this group: approximately 14% higher for MenACWY and 20% higher for MenB vaccines. The average VCI score for opinion leaders was 43.0 and the average VCI score for non-opinion leaders was 38.4 (Table 2). We also note greater perceptions of vaccine importance among opinion leaders for MenACWY, MenB, HPV, HBV, Hib, PCV13, and PPSV23. Varicella vaccine was received by approximately 5% fewer opinion leaders compared to non-opinion leaders, however this difference was not statistically significant.

Using a cut threshold of α = 0.10, statistically significant sociodemographic and health correlates of opinion leadership at the bivariate level were entered into a multivariate model predicting opinion leadership (Table 3). PrEP use and positive HIV status have a mutually exclusive relationship, meaning they could not be incorporated in the same regression model. A model with the statistically significant covariates was run on only HIV positive participants and PrEP was not found to be a predictor for opinion leadership membership, controlling for the other variables. We therefore excluded PrEP in following analyses in order to utilize the whole population, HIV positive, and HIV negative participants. Respondents who reported previously having an STI had greater odds of being an opinion leader compared to those without history of an STI (aOR = 2.02; 95% CI: 1.39–6.13). Those who identified as a race/ethnicity other than White, Black, or Hispanic (aOR = 2.91; 95% CI: 1.41–6.29), and Black/African Americans (aOR = 2.59; 95% CI: 1.15–5.80) had greater odds of being an opinion leader compared to White respondents. High income participants were more likely to be classified as an opinion leader compared to those with lower incomes (aOR = 2.36; 95% CI: 1.06–5.28).

### 3.3. Logistic Regression Results 

We ran 15 additional logistic regression models to test whether vaccination uptake and importance survey items predicted opinion leadership (Table 4). Two models were run, one with only the vaccine uptake or importance item and one controlled for variables that were statistically significant in the opinion leadership membership multivariable model (Table 3). For the unadjusted models, uptake of MenACWY (OR = 1.92; 95% CI: 1.13–3.25) and MenB (OR = 3.09; 95% CI: 1.77–5.41) were associated with opinion leadership. Perceiving HPV (OR = 1.86; 95% CI: 1.03–3.36), MenACWY (OR = 2.56; 95% CI: 1.44–4.56), Men B (OR = 2.06; 95% CI: 1.18–3.56), HBV (OR = 3.44; 95% CI: 1.05–11.3), Hib (OR = 1.64; 95% CI: 0.99–2.72), PCV13 (OR = 2.03; 95% CI: 1.22–3.38), and PPSV23 (OR = 2.24; 95% CI: 1.34–3.74) vaccines as important were also predictive of opinion leadership. We found relative consistency between the two sets of models in regard to significant predictors with slight differences in the degree of prediction with most adjusted odds ratios being slightly lower than their unadjusted counterparts due to the variations explained by the other included covariates.

We conducted a sensitivity analysis considering “unsure” responses as missing instead of “no”, which resulted in one notable changes to the results. The new value for Hib vaccine importance decreased to OR: 1.11 (95% CI: 0.58, 2.13) and was no longer statistically significant (*p*-value = 0.75); although we note that the *p*-value of 0.05 in the original model only just met the threshold for significance. All other uptake and importance predictors with significant *p*-values in the original models resulted in increased ORs and consistent significant *p*-values during sensitivity testing.

## 4. Discussion

This study is among the first to examine vaccine opinion leadership among MSM, a group disproportionately impacted by vaccine preventable illness, including meningitis, HAV, HBV, and HPV [30,31,32]. Opinion leaders have an important role to play in the diffusion of innovations in health, including vaccination messaging. Our results demonstrate that approximately 13.5% of MSM met criteria for opinion leadership. 

Black/African American MSM were more likely to be classified as opinion leaders compared to their White counterparts. This is notable given the historical disparities in vaccination coverage among racial/ethnic minority groups compared to Whites [33,34,35,36,37]. These results are also somewhat counterintuitive given the documented skepticism regarding biomedical health interventions among Black/African American MSM [38,39,40,41]. One possible explanation for the favorable attitudes toward vaccination among Black MSM in this context is the intense outreach and engagement targeted to communities of color during the ongoing meningococcal outbreak in Southern California. Capitalizing on this association and establishing partnerships with minority community leaders may be especially important considering vaccination coverage disparities between racial/ethnic minority and white adults.

The majority of participants in our sample were classified as ‘high risk’ and opinion leaders were more likely to have been diagnosed with an STI compared to non-opinion leaders. MSM previously diagnosed with an STI may have more opportunities to interact with healthcare providers and to engage in conversations explicitly focused on sexual health compared to those who have never been diagnosed with an STI. Previous research with MSM demonstrates discomfort in discussing sexual practices with healthcare providers [42,43]. Our findings suggest that key partnerships—such as the co-location of health promotion activities, STI services, and immunization services—could be an effective strategy for increasing vaccination and other preventive health interventions among MSM. A greater percentage of HIV-positive participants were classified as opinion leaders compared to non-opinion leaders. Although these results were not statistically significant in multivariable analyses, they suggest the potential utility of engaging HIV-positive MSM in vaccination-related health promotion. Regular engagement with the healthcare system may enable these men to become aware of vaccine preventable disease outbreaks more quickly and to disseminate this information to other MSM. Vaccination recommendations are sometimes different for people living with HIV [44,45] so special education may be warranted. 

Past studies have established the efficacy of social-network level interventions with regard to HIV prevention [20,21,22]. Researchers have enlisted and trained opinion leaders to promote HIV prevention messages during conversation with other MSM, resulting in decreased HIV-risk behaviors (e.g., CAS) and increased HIV-protective behaviors (e.g., HIV testing, condom use) among their peers [20,21,22]. Through dialogue with members of their existing social networks, opinion leaders can utilize their established personal social influence to cultivate positive attitudes towards, increase intentions and self-efficacy surrounding, and normalize the discussion of HIV protective behaviors in their communities [21]. Our data provide needed insight on which factors are associated with opinion leadership in the context of a meningococcal disease outbreak. Opinion leaders were more likely than non-opinion leaders to have received MenACWY and MenB vaccination, as well as to consider these and certain other vaccines as important. These results suggest that MSM who have been vaccinated may be key partners in influencing others to receive vaccinations during an outbreak. 

Given the widespread social media (i.e., Facebook, Twitter, Instagram) and geosocial networking app (e.g., Hornet, Grindr, Scruff) use by MSM it is important to consider technology-based vaccination promotion interventions. Opinion leadership interventions have been updated in recent years to incorporate new technologies. For example, in studies in Los Angeles, Lima, and Taiwan, trained opinion leaders utilized Facebook to disseminate HIV prevention messages to other internet-using MSM [19,23,46]. Though results varied by study, participants who received these interventions were more likely to request HIV testing [23,46], receive HIV tests [19,23,46], and consistently use condoms during anal sex with sex partners met online [19] compared to control group participants. In one of these studies, Young and colleagues (2014) found the social media-based opinion leadership intervention to be feasible and acceptable among the subject pool comprised of primarily Black/African American and Latino MSM [47]. At least one study that incorporates popular opinion leadership engagement in online chat rooms for the purpose of HIV prevention is currently under evaluation; results have not yet been published [48]. The efficacy of social media-based opinion leadership interventions demonstrated by previous HIV prevention studies, suggests the potential for sending and receiving health messages via the internet for the purpose of increasing vaccine uptake.

Our study findings should be interpreted in the context of some limitations. Our data were cross-sectional, and findings should be interpreted as correlational. Although we used venue-based sampling, a strategy that would theoretically result in greater generalizability, this sample may not be representative of all MSM in Los Angeles County. For example, MSM under 18 years of age and those who did not attend gay-identified venues during the recruitment period were not sampled. In addition, vaccination outcomes were measured using self-report and therefore may not represent actual vaccination behavior due to recall, response, and social desirability bias. Because our study was a rapid response to the unfolding events of the IMD outbreak, we were not able to verify vaccination status through a third party. Future studies should seek to verify immunization status in collaboration with health care providers or with data from immunization registries. 

## 5. Conclusions

We examined the characteristics of opinion leaders, including their uptake and attitudes toward vaccinations, within the context of a meningococcal disease outbreak in Los Angeles, California. Overall, respondents who identified as Black/African American or as a race/ethnicity other than White, Black, or Hispanic, and those with a history of an STI (other than HIV) were more likely to be opinion leaders. In addition, reported receipt of MenACWY and MenB vaccines were associated with opinion leadership among MSM. Our research builds upon the existing opinion leadership literature by identifying critical characteristics of opinion leaders in the MSM community. This work may be particularly useful in developing and prioritizing future vaccine messages that may be rapidly diffused through MSM communities.

## Figures and Tables

**Table 1:** Percentages of opinion leadership (yes/no) by sociodemographic, health, and technology use covariates using chi-square significance tests.

Variable	Non-Opinion Leader (*N* = 450)	Opinion Leader (*N* = 70)	Total (*N* = 520)	*p*-Value
**Sociodemographics**				
Age (in Years)	33.1 (32.2, 34.1)	34.4 (32.3, 36.6)	33.3 (32.4, 34.1)	0.33
18–24	21.6	11.4	20.4	0.22
25–44	63.0	72.9	64.2	
45–64	14.7	14.3	14.6	
65+	0.7	1.4	0.8	
Race/Ethnicity				
White	36.9	26.1	35.4	0.03 *
Black	14.1	21.7	15.1	
Hispanic	33.6	26.1	32.5	
Other	15.4	26.1	17.0	
Education (BA or more)	50.6 (45.9, 55.2)	57.1 (45.3, 69.0)	51.3 (47.0, 55.7)	0.31
Employed Full-time	61.7 (57.2, 66.3)	67.1 (55.9, 78.4)	62.4 (58.2, 66.5)	0.39
Income (≥$20,000)	77.8 (73.9, 81.7)	87.1 (79.1, 95.2)	78.9 (75.4, 82.5)	0.07
Insured	88.3 (85.3, 91.3)	88.2 (80.4, 96.1)	88.3 (85.5, 91.1)	0.99
Type of Insurance				
Private	57.0	66.7	58.3	0.62
Medicaid/Medicare	25.7	15.3	24.3	
Other	5.7	6.1	5.7	
Resides in West Hollywood	33.2 (28.8, 37.6)	40.0 (28.2, 51.8)	34.0 (30.0, 38.1)	0.26
**Health**				
HIV-positive serostatus	10.6 (7.7, 13.5)	20.3 (10.6, 30.0)	11.9 (9.1, 14.7)	0.02 *
Uses Alcohol	86.9 (83.7, 90.0)	85.7 (77.3, 94.1)	86.7 (83.8, 89.7)	0.79
Uses Marijuana	50.8 (46.1, 55.4)	55.7 (43.8, 67.6)	51.5 (47.2, 55.9)	0.44
Other Drugs	47.9 (43.3, 52.5)	58.6 (46.7, 70.4)	49.2 (44.9, 53.4)	0.10
History of STI other than HIV	45.2 (40.6, 49.8)	64.3 (52.8, 75.8)	47.7 (43.4, 52.0)	<0.01 *
Sexual Partner				
Men Only	90.0	88.6	89.9	0.72
Men and Women	10.0	11.4	10.2	
No. Sexual Partners (6 months)				
0–5	72.7	68.6	72.2	0.77
6–10	13.2	15.7	13.5	
11+	14.1	15.7	14.3	
Receptive Condomless Anal Sex	51.2 (46.6, 55.8)	57.1 (45.3, 69.0)	51.9 (47.6, 56.3)	0.36
High Risk	65.2 (60.7, 69.8)	71.2 (60, 82.4)	65.9 (61.7, 70.1)	0.34
PrEP Use ^†^	15.9 (12.2, 19.5)	29.1 (16.7, 41.5)	16.0 (12.9, 19.2)	0.02 *
Multi Vitamin	49.8 (45.1, 54.4)	61.4 (49.7, 73.1)	51.3 (46.9, 55.6)	0.07
**Technology**				
Facebook Usage	86.6 (83.4, 89.8)	85.7(77.3, 94.1)	86.5 (83.5, 89.4)	0.83
Twitter Usage	39.9 (35.3, 44.4)	35.7 (24.2, 47.2)	39.3 (35.1, 43.5)	0.51
Daily Email Users	90.9 (88.2, 93.6)	92.8 (86.5, 99.0)	91.2 (88.7, 93.6)	0.62
Daily Phone Users	98.9 (97.9, 99.9)	98.6 (95.7, 100)	98.9 (97.9, 99.8)	0.82
Daily Social Media Users	89.4 (86.4, 92.3)	95.3 (90, 101)	90.2 (87.6, 92.8)	0.14
Used Internet for Vac. Info	57.6 (53.0, 62.1)	64.3 (52.8, 75.8)	58.5 (54.3, 62.7)	0.29
Used Internet for Vac. Location	46.2 (41.6, 50.8)	51.4 (39.4, 63.4)	46.9 (42.6, 51.2)	0.42

* Indicates significant difference at α = 0.05 level; ^†^ Calculated only for those who are HIV negative, *N* = 396 and 55; Note: Some percentages may not add to 100.0 due to rounding.

**Table 2:** Percentages of opinion leadership (yes vs. no) by vaccine uptake and importance.

Outcome	Non-Opinion Leader (*N* = 450)	Opinion Leader (*N* = 70)	*p*-Value
**Vaccination Uptake**			
MenACWY	24.7% (111)	38.6% (27)	0.01 *
MenB	14.4% (65)	34.3% (24)	< 0.01 *
**Vaccination Importance**			
HPV	64.4% (290)	77.1% (54)	0.04 *
MenACWY	54.9% (247)	75.7% (53)	< 0.01 *
MenB	54.9% (247)	71.4% (50)	<0.01 *
HAV	83.8% (377)	87.1% (61)	0.47
HBV	86.7% (390)	95.7% (67)	0.03 *
Flu	63.6% (286)	64.3% (45)	0.91
Hib	42.0% (189)	54.3% (38)	0.05 *
MMR	74.4% (335)	82.9% (58)	0.13
Td/Tdap	80.4% (362)	87.1% (61)	0.18
Varicella	70.7% (318)	65.7% (46)	0.40
Zoster	67.1% (302)	74.3% (52)	0.23
PCV13	41.1% (185)	58.6% (41)	< 0.01 *
PPSV23	38.7% (174)	58.6% (41)	< 0.01 * 0.0
Average VCI ^a^	38.4	43.0	<0.01 *

* Value significant at *α* = 0.05 level; ^a^ 2 sample *t*-test was performed.

**Table 3:** Multivariable logistic regression for opinion leadership membership.

Outcome	aOR	95% CI	*p*-Value
HIV status (ref = negative)	1.69	(0.82, 3.58)	0.16
Race (ref = White)			
Black	2.59	(1.15, 5.80)	0.02 *
Hispanic	1.22	(0.59, 2.50)	0.59
Other	2.91	(1.39, 6.13)	<0.01 *
Any STI (ref = No)	2.02	(1.14, 3.58)	0.02 *
Other Drug Use (ref = No)	1.35	(0.78, 2.33)	0.28
Multivitamin Use (ref = No)	1.54	(0.89, 2.67)	0.13
High Income (ref = ≤$20,000/year)	2.36	(1.06, 5.28)	0.04 *

* Value significant at α = 0.05 level; Ref = reference group.

**Table 4:** Multivariable logistic regression models for opinion leadership membership by vaccine outcome.

Outcome	OR	95% CI	*p*-Value	aOR ^a^	95% CI	*p*-Value
Vaccination Uptake						
MenACWY	1.92	(1.13, 3.25)	0.01 *	*1.94*	(1.11, 3.40)	0.02 *
MenB	3.09	(1.77, 5.41)	<0.01 *	*2.97*	(1.653, 5.34)	<0.01 *
Vaccination Importance						
HPV	1.86	(1.03, 3.36)	0.04 *	*1.88*	(1.02, 3.45)	0.04 *
MenACWY	2.56	(1.44, 4.56)	< 0.01 *	*2.33*	(1.28, 4.23)	<0.01 *
MenB	2.06	(1.18, 3.56)	<0.01 *	*1.84*	(1.03, 3.27)	0.04 *
HAV	1.31	(0.62, 2.76)	*0.47*	*1.18*	(0.55, 2.57)	*0.67*
HBV	3.44	(1.05, 11.3)	0.03 *	*3.32*	(0.99, 11.12)	0.05 *
Flu	1.03	(0.61, 1.75)	*0.91*	*0.95*	(0.55, 1.6)	*0.86*
Hib	1.64	(0.99, 2.72)	0.05 *	*1.73*	(1.01, 2.95)	0.05 *
MMR	1.66	(0.86, 3.20)	*0.13*	*1.58*	(0.81, 3.09)	*0.18*
Td/Tdap	1.65	(0.79, 3.45)	*0.18*	*1.51*	(0.71, 3.21)	*0.29*
Varicella	0.80	(0.47, 1.36)	*0.40*	*0.76*	(0.44, 1.34)	*0.35*
Zoster	1.42	(0.80, 2.51)	*0.23*	*1.41*	(0.78, 2.57)	*0.26*
PCV13	2.03	(1.22, 3.38)	< 0.01 *	*1.80*	(1.06, 3.05)	0.03 *
PPSV23	2.24	(1.34, 3.74)	< 0.01 *	*1.96*	(1.16, 3.34)	<0.01 *

* Value significant at *α* = 0.05 level; ^a^ Controlling for covariates significant at *α* = 0.05 at bivariate level: race/ethnicity, STI history, income.
